# Germline-mediated ubiquitous recombination in ScxCre male mice: Implications for tendon research

**DOI:** 10.1371/journal.pone.0353660

**Published:** 2026-07-13

**Authors:** Haiyin Li, Christopher L. Mendias, Chike Cao

**Affiliations:** 1 Department of Orthopaedics, University of Rochester Medical Center, Rochester, New York, United States of America; 2 Center for Musculoskeletal Research, University of Rochester Medical Center, Rochester, New York, United States of America; 3 Performance Medicine Institute, Phoenix, Arizona, United States of America; Shanghai Jiao Tong University, CHINA

## Abstract

Scleraxis (Scx), a basic helix-loop-helix (bHLH) transcription factor, is a primary marker for tendon and ligament lineages. Accordingly, mouse models utilizing Cre recombinase under the control of the Scx locus provide a powerful tool for conditional gene manipulation in tendon and ligament tissues. The constitutive ScxCre mouse line is widely used for tendon-specific genetic studies. In this study, we show that ScxCre exhibits substantial unintended off-target activity in the male germline, resulting in ubiquitous recombination of floxed alleles in all tissues of the offspring. Transmission of recombined LoxP alleles occurred independently of Cre inheritance, indicating that ScxCre-mediated recombination took place before meiosis in diploid male germ cells. In contrast, this off-target activity was not observed in female germline. These findings highlight a critical need for stringent parental sex selection when using ScxCre lines to ensure tissue-specific targeting and to avoid unintentional global gene deletion or transgene activation.

## Introduction

The Cre-loxP system is one of the most powerful research tools used in mouse genetic manipulation. It enables the control of gene expression in a spatio-temporal manner and allows for cell lineage tracing by turning on the expression of a reporter transgene. This system relies on the site-specific recombinase, Cre, derived from bacteriophage P1 and two 34-bp DNA recognition sequences known as loxP sites. The Cre recombinase recognizes these sites and catalyzes the recombination of DNA sequences flanked by them (called “floxed” gene). To exploit this system, a collection of Cre mouse lines has been generated in which Cre expression is controlled by tissue-specific promoters. Crossing these Cre mice with a strain bearing a floxed target results in DNA recombination in specific cell types, yielding tissue-specific knockouts or transgene expression. The reliability of this system is critically dependent on the specificity of the chosen promoter. However, since most genes are expressed across multiple tissues, concerns persist regarding the fidelity of the Cre-loxP system and the potential for “off-target” recombination [1–5].

Scx is a basic helix-loop-helix (bHLH) transcription factor that is persistently expressed in tendons and ligaments from early developmental through adulthood [6]. Scx plays a key role in tendon progenitor cell fate, specification, differentiation and maturation [7]. Homozygous Scx^-/-^ null mice exhibit a severe loss in intermuscular tendons and those responsible for all force-transmitting, which significantly limits the use of all paws and dorsal muscles [7]. To date, Scx remains the best-characterized and most widely used marker for the tendon lineage [6,8,9]. Consequently, genetic manipulation of the Scx locus provides a unique opportunity to target gene expression in tendon progenitors and mature tenocytes. The constitutive ScxCre transgenic mouse line, generated by BAC transgenesis [10], is a powerful tool for modulating gene expression during tendon/ligament development and pathology.

In this study, we demonstrate that ScxCre transgene leads to the germline deletion of loxP-flanked alleles when carried in the male germline, but not the female. Consequently, offspring of ScxCre-expressing males exhibit germline-recombined alleles and subsequent reporter gene activity ubiquitously across all tissues. Notably, even offspring that do not inherit the ScxCre transgene itself still possess the recombined alleles, indicating that Cre-medicated recombination in the male germline occurs in premeiotic diploid cells. Therefore, when ScxCre and a floxed allele are carried together in a male parent, ScxCre-mediated gene targeting loses its tissue specificity. To avoid unintended germline recombination, breeding strategies must be carefully designed; specifically, the ScxCre transgene should be introduced through the female parent to ensure the target alleles remain tissue-specific and to avoid the paternal germline leakage observed in male carriers.

## Materials and methods

### Experimental mouse models and breeding strategies

Animal husbandry and all animal procedures were performed in accordance with the protocols approved by the Institutional Animal Care and Use Committee at the University of Rochester Medical Center. All mice were group-housed on a reversed 12-hour light/dark cycle and provided with ad libitum access to food and water. Adult mice were euthanized by CO_2_ inhalation, and death was confirmed by cervical dislocation. Neonatal pups at postnatal day 0-4 (P0-P4) were euthanized by hypothermia on ice, immediately followed by removal of skin and internal organs for subsequent fixation for whole-mount X-gal staining and tail-tip collection for genotyping.

The transgenic ScxCre mouse line was generously provided by Dr. Ronen Schweitzer, and is now available from the Jackson Laboratory (Strain No: 032131). This line carries a BAC transgene in which the Scx promoter drives GFP/Cre expression. Although this line has been widely used in the field, to our knowledge no separate primary publication specifically describing its original generation and full validation has been published. The ScxCre line was first used in the original study [10] and the Ca_V_1.2-TS^fl/fl^ [11] mouse line has been described previously. C57BL/6J (Strain No: 000664), RlacZ^fl/fl^ (Strain No: 003474), Ai9^fl/fl^ (Strain No: 007909) strains were obtained from the Jackson Laboratory. All mice were on a C57BL/6J genetic background.

To trace ScxCre and Ca_V_1.2-TS mutant channel expression, male ScxCre;Ai9^fl/+^ mice were crossed with female Ca_V_1.2-TS^fl/fl^ mice. For examining recombination in testes and ovaries, ScxCre mice were crossed with RlacZ^fl/fl^ mice to generate ScxCre;RlacZ^fl/+^ progeny, which were subsequently backcrossed with male or female C57BL/6J mice.

### X-gal staining

Whole-mount X-gal (5-bromo-4-chloro-3-indolyl-β-D-galactopyranoside) staining was performed on neonatal mice between P0 and P4. Samples followed skin and internal organ removal were fixed in 4% paraformaldehyde on ice for 1 hour, washed three times with 1x PBS and incubated in X-gal staining solution (5mM potassium ferrocyanide, 5mM potassium ferricyanide, 1 mg/ml X-gal, 2mM MgCl2, 0.1% sodium deoxycholate, 0.2% IGEPAL CA-630) in the dark for 12 hours at 37 °C with gently shaking.

### Genotyping

Genomic DNA was extracted from ear punches of adult mice or tail tips of P0-P4 pups and PCR genotyping was performed using GoTag^®^ Master Mix (Promega, Cat. No. M7122). The PCR amplification program consisted of an initial denaturation at 94 ºC for 3 minutes, followed by 35 cycles of 94 ºC for 30 seconds, 61 ºC for 1 minute and 72 ºC for 1 minute, and a final cycle of 72 ºC for 5 minutes. DNA fragments were resolved on 2% agarose gel containing SYBR^TM^ Safe DNA Gel Stain (ThermoFisher, Cat. No. S33102) and visualized using a Bio-Rad gel documentation system. The primers used in this study are listed below:

ScxCre-F: 5’-GCAGAACCTGAAGATGTTCGC-3’
ScxCre-R: 5’-ACACCAGAGACGGAAATCCATC-3’
RosaWT-R: 5’-AAGGGAGCTGCAGTGGAGTA-3’
RosaWT-F: 5’-CCGAAAATCTGTGGGAAGTC-3’
Ai9(floxed)-F: 5’-CTGTTCCTGTACGGCATGG-3’
Ai9(floxed)-R: 5’-GGCATTAAAGCAGCGTATCC-3’
Ai9^Δ^ (recombined)-F: 5’-CGTGCTGGTTATTGTGCTGT-3’
Ai9^Δ^ (recombined)-R: 5’-GCAGGTCGAGGGACCTAATA-3’
RlacZ WT-R: 5’-GGAGCGGGAGAAATGGATATG-3’
RlacZ common-F: 5’-AAAGTCGCTCTGAGTTGTTAT-3’
RlacZ(floxed)-R: 5’-GCGAAGAGTTTGTCCTCAACC-3’

### Single-cell RNA sequencing analysis

To determine which testicular cell types express endogenous Scx, we reanalyzed a published single-cell RNA-sequencing atlas of the adult mouse testis generated by Drop-seq (Gene Expression Omnibus accession GSE112393) [12]. The processed digital gene expression matrix and the accompanying per-cell attribute table were obtained from the series record, and each cell was assigned to its published cell type using the author-provided annotations, comprising 34,633 cells across four germ cell populations (spermatogonia, spermatocytes, round spermatids, and elongating spermatids) and seven somatic populations (Sertoli, Leydig, myoid, endothelial, macrophage, innate lymphoid, and an unannotated mesenchymal population). Analysis was performed in Python using Scanpy (v1.12). Transcript counts were normalized to 10,000 per cell and log transformed. Scx expression was quantified per cell type as the percentage of cells with at least one detected transcript and as mean log normalized expression, and was visualized on a Uniform Manifold Approximation and Projection (UMAP) embedding computed from the top 2,000 highly variable genes and the first 30 principal components. Sertoli cell identity was confirmed with Sox9 and Wt1, and germ cell identity was confirmed with Ddx4, Dazl, Zbtb16, Sycp3, and Prm1. Because the original study enriched for rare cell types, cell type proportions in this dataset do not reflect their in vivo abundance; therefore, all comparisons here are limited to within-cell-type expression frequency, which is not affected by this enrichment strategy.

## Results

### Detection of recombined loxP alleles in Cre-negative offspring of ScxCre mice

**Fig 1:**
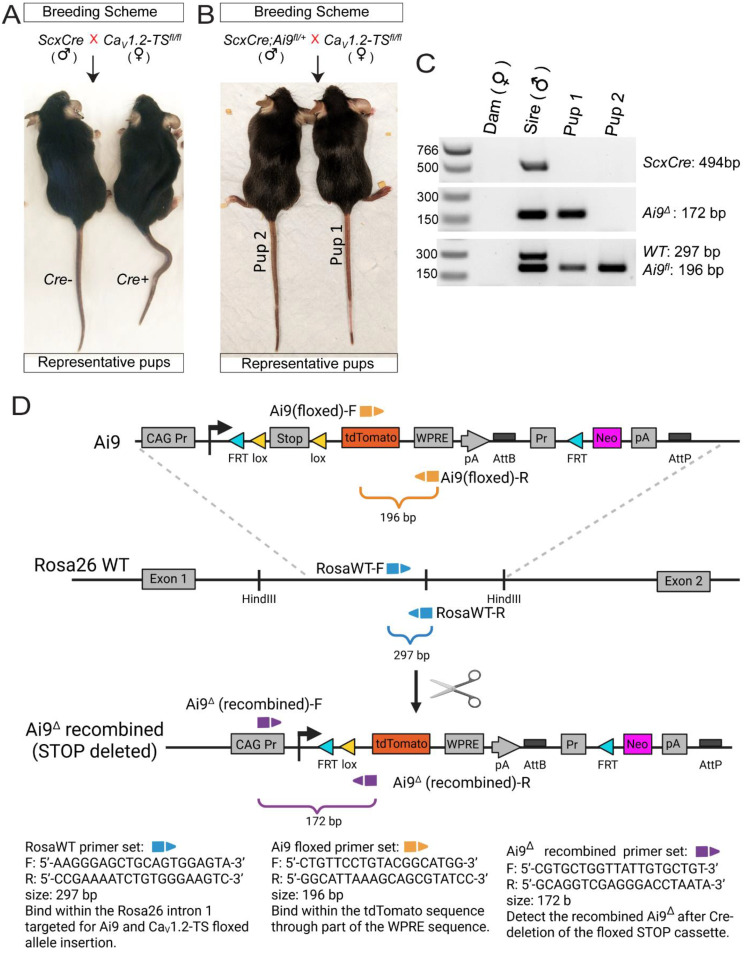
Breeding with ScxCre males causes germline recombination and unexpected recombined alleles in Cre-negative offspring. (A) Representative images of offspring from male ScxCre mice crossed with female Ca_V_1.2-TS^fl/fl^ mice. ScxCre;Ca_V_1.2-TS^fl/+^ mice exhibit the wavy-tail phenotype compared with ScxCre-negative littermate controls. (B) Representative progeny generated by breeding male ScxCre;Ai9^fl/+^ mice with female Ca_V_1.2-TS^fl/fl^ mice, showing germline recombination of the Ai9 reporter allele in offspring lacking inherited ScxCre. (C) PCR genotyping of ear-punch DNA from the dam, sire, and two representative pups. The top panel detects ScxCre (494 bp). The middle panel detects the Cre-recombined Ai9Δ allele (172 bp). The bottom panel detects the intact Rosa26 wild-type allele (WT, 297 bp) and the unrecombined Ai9 floxed allele (196 bp). Because the Ca_V_1.2-TS floxed transgene is also inserted into the Rosa26 locus, the intact Rosa26 WT allele is disrupted in Ca_V_1.2-TS^fl/fl^ mice; therefore, no WT band is expected in the dam or in offspring lacking an intact Rosa26 WT allele. (D) Schematic diagram adapted from Madisen et al. [14] illustrating the primer-binding strategy used to distinguish the Rosa26 WT, Ai9^fl^, and Cre-recombined Ai9^Δ^ alleles.

To assess the effects of tendon-specific Ca_V_1.2 gain-of-function in vivo, as previously reported [13], we crossed male ScxCre mice with female Ca_V_1.2-TS^fl/fl^ mice. The offspring with genotype ScxCre;Ca_V_1.2-TS^fl/+^ developed wavy tails with 100% penetrance in both male and female pups (Fig 1A), a phenotype caused by abnormal tail tendon formation induced by expression of the mutant Ca_V_1.2-TS channel [13]. Unexpectedly, when tdTomato was used as a lineage-tracing reporter by crossing male ScxCre;Ai9^fl/+^ with female Ca_V_1.2-TS^fl/fl^ mice, we observed reporter activation in Cre-negative offspring: Pup1 displayed a normal, straight tail, consistent with the absence of ScxCre-driven Ca_V_1.2-TS expression, but nevertheless showed ubiquitous tdTomato expression, visible as a red-pink hue in the tail, limbs and ears (Fig 1B, Pup 1). Further, PCR genotyping of genomic DNA (gDNA) from ear biopsies confirms the absence of the ScxCre transgene in Pup 1, despite the presence of the recombined tdTomato allele (Ai9^Δ^) (Fig 1C). These findings indicate that germline recombination occurred in the male germline prior to transmission, resulting in paternal inheritance of a pre-recombined alleles.

### Verification of germline recombination in male, but not female, ScxCre mice

**Fig 2:**
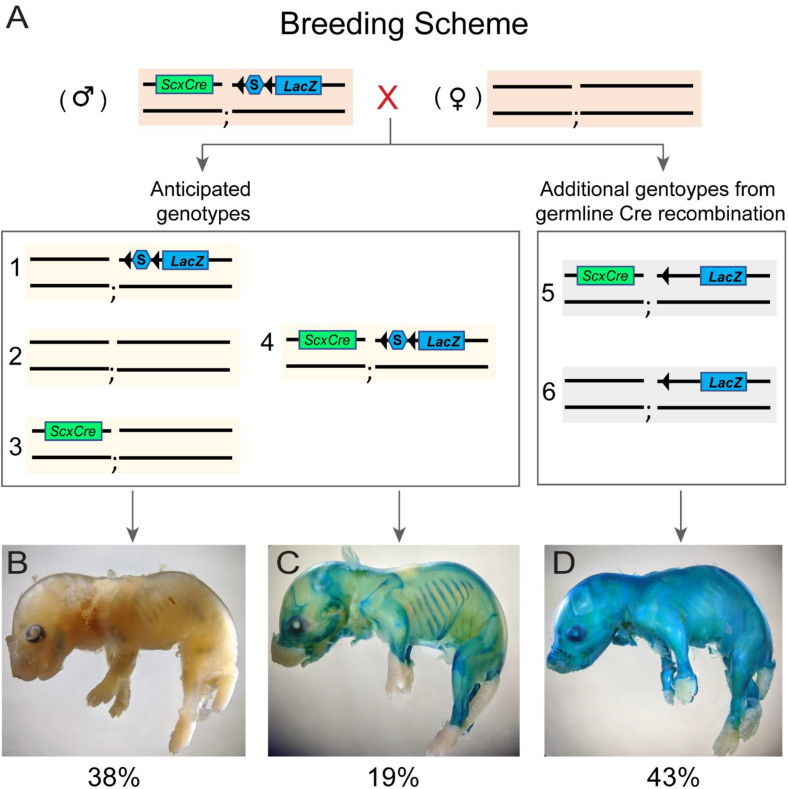
Germline recombination of the RlacZ floxed allele in male ScxCre mice. (A) Schematic showing the anticipated genotypes (genotypes 1, 2, 3, and 4) and the additional genotypes (genotypes 5 and 6) generated by germline Cre recombination in crosses between male ScxCre;RLacZ^fl/+^ mice and female C57BL/6J mice. (B-D) Representative whole-mount X-gal staining images of offspring from this cross, showing unstained, tissue-restricted, and diffusely stained patterns, respectively. Blue staining indicates β-galactosidase expression following Cre-mediated recombination of the RlacZ reporter allele. The percentages under each image indicate the frequency of each staining pattern among offspring. In total, 21 pups from 4 independent litters were analyzed.

To independently validate ScxCre germline recombination using a different floxed allele, we crossed male ScxCre;RLacZ^fl/+^ mice with female C57BL/6J mice. The presence and absence of Cre and RlacZ transgenes in the generated offspring were determined by PCR. To detect RlacZ transgene recombination and the resulting β-galactosidase activity, whole-mount X-gal staining was performed on neonatal mice (P0-P4). Whereas standard Mendelian inheritance predicts only four possible genotypes (Fig 2A, genotypes 1, 2, 3 and 4), germline recombinaton generates tow additional possible genotypic outcomes (Fig 2A, genotypes 5 and 6). As expected, no β-galactosidase activity was observed in 38% of the offspring, corresponding to genotypes 1, 2, and 3 (Fig 2B). In 19% of the offsping, Cre activity was appropriately restricted to the tendons and ligaments of the limbs, ribs, and craniofacial sutures, corresponding to genotypes 4 (Fig 2C). However, approxiamately 43% of the offspring displayed widespread X-gal staining throughout the body (Fig 2D), indicating germline recombination. Notably, within this “whole-body” staining group, 5 of 9 pups were Cre-positive (genotype 5), while 4 of 9 pups had not inherited the ScxCre transgene (genotype 6). This distribution suggests that recombination occurred during gametogenesis prior to the completion of meiosis. The frequency of each observed genetoype is summarized in Table 1. Collectively, these data show that a germline-recombined gentoype occurred in 43% of all offspring sired by a ScxCre-positive male.

**Table 1:** Frequencies of offspring genotypes by parental Cre source.

Cre-bearing parent	Offspirng Genotype					
	Type 1	Type 2	Type 3	Type 4	Type 5	Type 6
**Cre in sire**	0/21	6/21	2/21	4/21	5/21	4/21
**Cre in dam**	7/22	4/22	4/22	7/22	0/22	0/22

Note: Total 21 pups from 4 independent litters for Cre in Sire, and 22 pups from 4 litters for Cre in Dam were analyzed.

To determine whether the ScxCre transgene also triggers germline recombination when transmitted maternally, we cross female ScxCre;RLacZ^fl/+^ mice with male C57BL/6J mice. In contrast to the paternal crosses, we observe no instances of widespread X-gal staining in the resulting offspring. Furthermore, PCR genotyping confirmed that β-galactosidase activity remained strictly dependent on the inheritance of the ScxCre transgene, with no evidence of pre-meiotic recombination. The observed genotype frequencies for this maternal inheritance strategy are summarized in Table 1.

### scRNA-seq analysis reveals minimal endogenous Scx expression in the male germline

**Fig 3:**
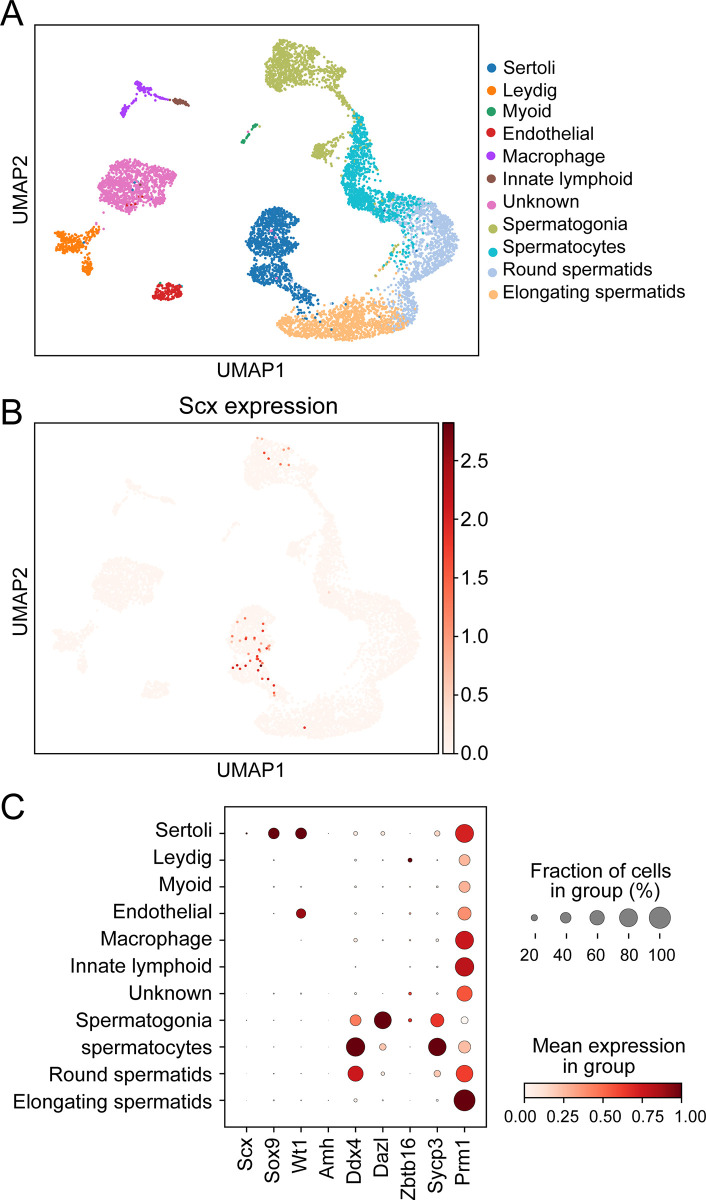
Endogenous Scx is expressed in testicular somatic cells and is minimal in the male germline. Reanalysis of a published adult mouse testis single-cell RNA-sequencing dataset, using the author-provided cell type annotations. (A) UMAP embedding of testicular cells colored by cell type, resolving the germ cell trajectory (spermatogonia, spermatocytes, round spermatids, and elongating spermatids) and the somatic cell populations (Sertoli, Leydig, myoid, endothelial, macrophage, and innate lymphoid cells), along with an unannotated mesenchymal population. (B) Scx expression overlaid on the UMAP embedding, showing signal primarily in the Sertoli cell cluster. (C) Dot plot of Scx and reference cell type markers across cell types. Dot size indicates the fraction of cells in each group expressing the gene, and color indicates mean expression scaled to the maximum across cell types. Sertoli markers (Sox9, Wt1) and germ cell markers (Ddx4, Dazl, Zbtb16, Sycp3, Prm1) support cluster identity.

To determine whether endogenous Scx is expressed in the male germline, we reanalyzed a published single-cell RNA-sequencing dataset of adult mouse testis [12]. The UMAP embedding resolved the expected testicular cell populations, including spermatogonia, spermatocytes, round spermatids, elongating spermatids, and multiple somatic cell types (Fig 3A). Cell identities were supported by expression of established marker genes, including Ddx4, Dazl, Zbtb16, Sycp3, and Prm1 for germ cells, and Sox9 and Wt1 for Sertoli cells. Overlay of Scx expression on the UMAP showed that Scx-positive cells were localized primarily to the Sertoli cell cluster, with only rare transcript detection in germ cell populations (Fig 3B). Quantification of Scx expression across annotated cell types was visualized using a dot plot (Fig 3C). Scx expression was detected in 3.2% of Sertoli cells, compared with 0.7% of spermatogonia, 0.3% of spermatocytes, 0.04% of round spermatids, and 0.09% of elongating spermatids, representing only 0.2% of germ cells overall. These findings indicate that endogenous Scx expression is not appreciably expressed in the male germline and is instead primarily detected in a small subset of testicular somatic cells.

## Discussion

Scx is widely regarded as a well-established marker for tendons and ligaments [6,7,15]. To date, the ScxCre mouse line generated by Dr. Ronen Schweitzer and colleagues remains an essential tool for manipulating gene expression during tendon/ligament development and disease pathology [10]. However, endogenous Scx expression is also present in other tissues, including testes [15–17], which raises the potential for germline recombination. In this study, we demonstrate that when ScxCre is transmitted paternally, 43% of offspring carry recombined RlacZ alleles throughout the body, regardless of the tissue-specific Scx expression or the inheritance of the ScxCre transgene itself. Although recombination efficiency may vary depending on the specific floxed target, this germline recombination was notably absent in the offspring of ScxCre females. This is consistent with previous findings that Scx mRNA is not detected in the ovary [16]. Taken together, these data indicate that using ScxCre females as the Cre-carrying parent is the preferred breeding strategy to preserve tissue-specific gene manipulation and avoid unintended global recombination.

Strong evidence indicates that endogenous Scx expression in the testis is primarily restricted to Sertoli cells within the seminiferous tubules [15–17]. Consistent with these reports, our reanalysis of a published adult mouse testis single-cell RNA-sequencing dataset [12] showed that Scx-positive cells were localized primarily to the Sertoli cell cluster, whereas Scx expression was rare across germ cell populations. Because Sertoli cells are somatic support cells that provide nutrients and structural support to developing germ cells, rather than components of the germline itself [18,19], these data argue against appreciable endogenous Scx expression in the male germline. Therefore, the paternal germline recombination observed in the ScxCre mouse line is unlikely to be explained by native Scx activity in germ cells. Instead, it may reflect properties of the ScxCre transgene itself.

The ScxCre mouse line was generated via BAC (bacterial artificial chromosome) transgenesis [10], in which Cre recombinase was introduced into a BAC containing large mouse genomic sequences from the Scx locus. BAC transgenic approaches can be advantageous because the large genomic insert may contain many distal regulatory elements needed to approximate endogenous gene expression [20]. However, unlike knock-in strategies, BAC transgenes are generally inserted randomly into the genome and may be present in variable copy numbers. As a result, BAC-driven Cre expression can be influenced by insertion-site effects, copy-number effects, or missing/altered regulatory elements, and therefore may not fully recapitulate the endogenous expression pattern of the target gene [20], such as Scx. In contrast, knock-in approaches place Cre or CreERT2 into a defined endogenous locus, which may more closely preserve native regulatory control, although knock-in designs can disrupt the targeted allele or alter gene expression depending on the insertion strategy. Thus, the ectopic male germline recombination observed in the ScxCre line may reflect BAC transgene-specific expression properties rather than endogenous Scx activity in germ cells. This distinction is important for interpreting the current findings and for designing breeding strategies when using BAC-derived Cre lines.

Germline recombination in many Cre lines may go unrecognized because detecting such events often requires a two-generation breeding scheme. In this approach, F1 offspring (Cre^+^; reporter^fl/+^) are crossed with wild-type mice, and reporter expression is then evaluated in the F2 generation. To prevent unintended recombination and the subsequent misinterpretation of phenotypes, rigorous characterization of any Cre line is essential. First, Cre activity outside the target tissue, especially in the testes or ovaries, should be validated by crossing the Cre-driver mouse with a floxed reporter and examining the offspring. Second, for transgene expression studies, breeding strategies should avoid using a single parent that carries both the Cre and the floxed allele, in order to prevent the co-inheritance of these elements. Finally, in conditional knockout studies, additional PCR genotyping using primers spanning the floxed region is important to detect recombined alleles. Together, these precautions are critical for identifying ectopic recombination and ensuring that observed phenotypes are truly tissue-specific.

One limitation of this study is that the ScxCre line used here is an established BAC transgenic line, and we did not perform transgene integrity mapping, copy-number analysis, insertion-site characterization, or comparisons across independent founder lines. Therefore, we cannot determine whether the observed male germline recombination reflects BAC transgene-specific properties, insertion-site effects, or other regulatory mechanisms. Nevertheless, our findings demonstrate that this widely used ScxCre line can mediate male germline recombination under the breeding conditions examined.

## Supporting information

S1 File Raw images. (PDF)
