# Supplementary figures and images for: Germline-mediated ubiquitous recombination in ScxCre male mice: Implications for tendon research
Source: PLoS One. 2026 Jul 13;21(7):e0353660. doi: 10.1371/journal.pone.0353660 (PMC13362121; doi:10.1371/journal.pone.0353660)

ScxCre

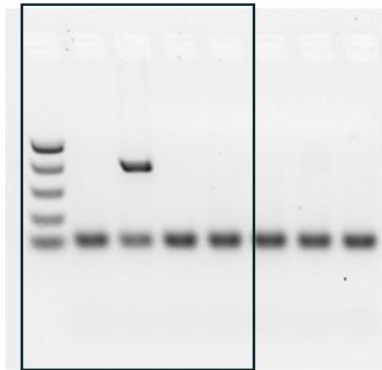

Ai9<sup>Δ</sup>

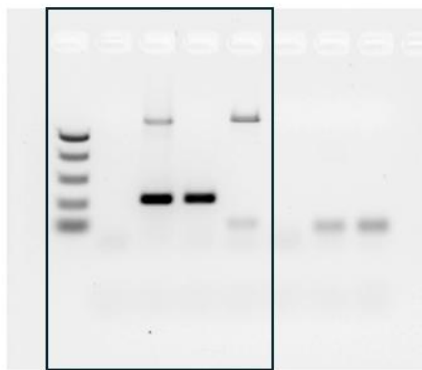

RosaWT & Ai9<sup>fl</sup>

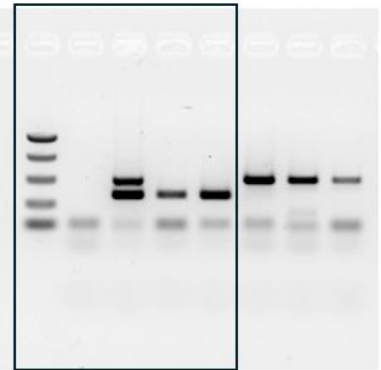

Supplement: S1 File — (PDF) [file pone.0353660.s001.pdf]
