# Supplementary material for: Germline-mediated ubiquitous recombination in ScxCre male mice: Implications for tendon research
Source: PLoS One. 2026 Jul 13;21(7):e0353660. doi: 10.1371/journal.pone.0353660 (PMC13362121; doi:10.1371/journal.pone.0353660)
Supplement: Attachment — Submitted filename: Response to Reviewers.docx [file pone.0353660.s003.docx]

**PONE-D-26-18919**

**Germline-mediated ubiquitous recombination in ScxCre male mice: implications for tendon research**

**PLOS One**

**Response to the Editor and Reviewers for PLOS ONE Submission**

We are most grateful to you and the reviewers for the careful assessment of this manuscript and providing insightful comments. In response to the reviewers’ comments, we revised the manuscript. Revisions in the manuscript are highlighted in yellow. The Reviewers’ suggestions and queries are reproduced verbatim in the “Response to reviewers” file, with our responses in **bold**.

**Reviewer #1**: This manuscript reports an important finding for researchers conducting similar studies using ScxCre mice and related Cre-loxP systems. The observation that paternal transmission of ScxCre can lead to germline-mediated ubiquitous recombination has substantial practical implications. In this sense, the manuscript has clear value, as it may help prevent misinterpretation of experimental results and improve the efficiency and reliability of research in this field.

I also appreciate that the manuscript does not merely report an unexpected phenomenon, but places it in a broader methodological context by discussing possible causes and proposing practical breeding strategies to avoid unintended germline recombination.

However, I believe that the following points would benefit from further clarification.

First, the discussion of BAC transgenesis is important for interpreting the present findings, but this approach may not be familiar to all readers. It would therefore be helpful if the authors could provide a slightly more detailed explanation of the characteristics and limitations of BAC transgenic approaches, particularly in comparison with other genetic engineering strategies such as knock-in approaches.
Second, BAC transgenesis can occasionally result in partial integration, rearrangement, copy-number variation, or insertion-site-dependent effects, which may alter expression specificity. While the authors’ interpretation of ectopic/leaky germline Cre activity is plausible and likely correct, clarification regarding whether transgene integrity or founder-specific effects have been evaluated would further strengthen the manuscript.

**Thank you for this insightful comment. We agree that additional discussion of BAC transgenesis and its limitations is important for interpreting our findings. We have revised the Discussion to provide more background on BAC transgenic approaches, including their advantages and limitations compared with knock-in strategies. Specifically, we now explain that BAC transgenes contain large genomic regulatory regions and can support tissue-relevant expression, but they typically integrate randomly and may be affected by insertion-site effects, copy-number variation, partial integration, rearrangement, or incomplete regulatory control.**

**We also agree that transgene integrity and founder-specific effects are important considerations. However, the ScxCre line used in this study was not generated by our group; it was an established mouse line originally generated by Blitz et al. and is now available from The Jackson Laboratory. Therefore, we were not able to evaluate founder-specific effects or compare independent founder lines. We did not perform additional transgene integrity mapping, copy-number analysis, or insertion-site characterization in the present study. In addition, we added new data in Figure 3 by reanalyzing a published single-cell RNA sequencing dataset of adult mouse testis, which showed that Scx is not expressed in the germline cell populations. We have clarified this limitation in the revised Discussion. Nevertheless, our data demonstrate that the established ScxCre line used by the field can cause male germline recombination under the breeding conditions tested, regardless of whether this reflects endogenous Scx regulation or BAC transgene-specific effects.**

**scRNA-seq analysis reveals minimal endogenous Scx expression in the male germline**

To determine whether endogenous Scx is expressed in the male germline, we reanalyzed a published single-cell RNA-sequencing dataset of adult mouse testis [12]. The UMAP embedding resolved the expected testicular cell populations, including spermatogonia, spermatocytes, round spermatids, elongating spermatids, and multiple somatic cell types. Cell identities were supported by expression of established marker genes, including Ddx4, Dazl, Zbtb16, Sycp3, and Prm1 for germ cells, and Sox9 and Wt1 for Sertoli cells. Overlay of Scx expression on the UMAP showed that Scx-positive cells were localized primarily to the Sertoli cell cluster, with only rare transcript detection in germ cell populations. Quantification across annotated cell types showed Scx expression in 3.2% of Sertoli cells, compared with 0.7% of spermatogonia, 0.3% of spermatocytes, 0.04% of round spermatids, and 0.09% of elongating spermatids, corresponding to only 0.2% of germ cells overall. These findings indicate that endogenous Scx expression is not appreciably expressed in the male germline and is instead primarily associated with testicular somatic cells.

**Figure 3. Endogenous scleraxis is expressed in testicular somatic cells and is minimal in the male germline.** Reanalysis of a published adult mouse testis single-cell RNA-sequencing dataset, using the author-provided cell type annotations. (A) UMAP embedding of testicular cells colored by cell type, resolving the germ cell trajectory (spermatogonia, spermatocytes, round spermatids, and elongating spermatids) and the somatic cell populations (Sertoli, Leydig, myoid, endothelial, macrophage, and innate lymphoid cells), along with an unannotated mesenchymal population. (B) Scx expression overlaid on the UMAP embedding, showing signal primarily in the Sertoli cell cluster. (C) Dot plot of Scx and reference cell type markers across cell types. Dot size indicates the fraction of cells in each group expressing the gene, and color indicates mean expression scaled to the maximum across cell types. Sertoli markers (Sox9, Wt1) and germ cell markers (Ddx4, Dazl, Zbtb16, Sycp3, Prm1) support cluster identity.

Discussion

…

The ScxCre mouse line was generated via BAC (bacterial artificial chromosome) transgenesis [10], in which Cre recombinase was introduced into a BAC containing large mouse genomic sequences from the Scx locus. BAC transgenic approaches can be advantageous because the large genomic insert may contain many distal regulatory elements needed to approximate endogenous gene expression [20]. However, unlike knock-in strategies, BAC transgenes are generally inserted randomly into the genome and may be present in variable copy numbers. As a result, BAC-driven Cre expression can be influenced by insertion-site effects, copy-number effects, or missing/altered regulatory elements, and therefore may not fully recapitulate the endogenous expression pattern of the target gene [20], such as Scx. In contrast, knock-in approaches place Cre or CreERT2 into a defined endogenous locus, which may more closely preserve native regulatory control, although knock-in designs can disrupt the targeted allele or alter gene expression depending on the insertion strategy. Thus, the ectopic male germline recombination observed in the ScxCre line may reflect BAC transgene-specific expression properties rather than endogenous Scx activity in germ cells. This distinction is important for interpreting the current findings and for designing breeding strategies when using BAC-derived Cre lines.

…

One limitation of this study is that the ScxCre line used here is an established BAC transgenic line, and we did not perform transgene integrity mapping, copy-number analysis, insertion-site characterization, or comparisons across independent founder lines. Therefore, we cannot determine whether the observed male germline recombination reflects BAC transgene-specific properties, insertion-site effects, or other regulatory mechanisms. Nevertheless, our findings demonstrate that this widely used ScxCre line can mediate male germline recombination under the breeding conditions examined.

**Reviewer #2:** In this manuscript, the authors demonstrate that ScxCre exhibits significant off-target recombination activity in the male germline, leading to ubiquitous recombination of floxed alleles in offspring independently of Cre inheritance. The study further shows that this phenomenon is specific to male transmission and is not observed upon maternal inheritance of ScxCre. Overall, the experiments are well designed and the data support the main conclusions.

This work is important for the field as it provides a critical technical caution for investigators using ScxCre lines. The finding that germline recombination can occur prior to meiosis in diploid germ cells has direct implications for breeding strategy design and interpretation of conditional knockout and lineage-tracing experiments. In particular, the study highlights the necessity of strict parental sex selection to avoid unintended global recombination events, which is highly relevant for the Cre-LoxP user community.

Minor comments:

1. In Figure 1C, three PCR products are shown for the Ai9 allele (WT, floxed, and recombined). The WT PCR shown for the Ai9 locus presumably amplifies the Rosa26 wild-type allele. If so, it is unclear why no WT band is observed in the dam and pups 1 and 2. The authors should clarify the expected genotypes and banding patterns for WT, heterozygous Ai9, and recombined alleles, as well as the primer binding strategy.
**Thank you for pointing this out. We agree that the expected banding pattern required clarification. The WT PCR product shown for the Ai9 allele detects the intact Rosa26 wild-type allele. In the breeding strategy used for Figure 1C, male ScxCre;Ai9^fl/+^ mice were crossed with female Ca_V_1.2-TS ^fl/fl^ mice. Because the floxed Ca_V_1.2-TS transgene is also inserted into the Rosa26 locus, the Rosa26 wild-type allele is disrupted in the dam and in offspring inheriting the Ca_V_1.2-TS allele. Therefore, no Rosa26 WT band is expected in the Ca_V_1.2-TS ^fl/fl^ dam or in pups carrying only Rosa26-targeted transgenic alleles rather than an intact Rosa26 WT allele. We have revised figure legend to clarify the expected genotypes, the banding patterns for WT, Ai9 floxed, and Ai9Δ recombined alleles, and the primer-binding strategy.**

**Figure 1. Breeding with ScxCre males causes germline recombination and unexpected recombined alleles in Cre-negative offspring.** (A) Representative images of offspring from male ScxCre mice crossed with female Ca_V_1.2-TS^fl/fl^ mice. ScxCre;Ca_V_1.2-TS^fl/+^ mice exhibit the wavy-tail phenotype compared with ScxCre-negative littermate controls. (B) Representative progeny generated by breeding male ScxCre;Ai9^fl/+^ mice with female Ca_V_1.2-TS^fl/fl^ mice, showing germline recombination of the Ai9 reporter allele in offspring lacking inherited ScxCre. (C) PCR genotyping of ear-punch DNA from the dam, sire, and two representative pups. The top panel detects ScxCre (494 bp). The middle panel detects the Cre-recombined Ai9Δ allele (172 bp). The bottom panel detects the intact Rosa26 wild-type allele (WT, 297 bp) and the unrecombined Ai9 floxed allele (196 bp). Because the Ca_V_1.2-TS floxed transgene is also inserted into the Rosa26 locus, the intact Rosa26 WT allele is disrupted in Ca_V_1.2-TS^fl/fl^ mice; therefore, no WT band is expected in the dam or in offspring lacking an intact Rosa26 WT allele. (D) Schematic diagram adapted from Madisen et al. [14] illustrating the primer-binding strategy used to distinguish the Rosa26 WT, Ai9^fl^, and Cre-recombined Ai9^Δ^ alleles.

2. In the Methods, the origin of the ScxCre line is cited as Ref. 10 however, this reference does not provide a primary description of the generation of the ScxCre mouse line, only states that it will be described elsewhere. Please include the appropriate original reference describing the generation and validation of this transgenic line.
**Thank you for this comment. To our knowledge, a stand-alone primary publication describing the original generation and validation of the specific ScxCre line used in this study has not been published. The line was originally provided by Dr. Ronen Schweitzer and is now available from The Jackson Laboratory as C57BL/6-Tg(Scx-GFP/cre)1Stzr/J, JAX stock no. 032131. The Jackson Laboratory lists the transgene as Tg(Scx-GFP/cre)1Stzr and describes it as a BAC transgene in which the Scx** **promoter drives a GFP/Cre sequence. We have revised the Methods section to clarify the source of the ScxCre line and to avoid implying that Ref. 10 provides a complete primary description of its generation.**

The transgenic ScxCre mouse line was generously provided by Dr. Ronen Schweitzer, and is now available from the Jackson Laboratory (Strain No: 032131). This line carries a BAC transgene in which the Scx promoter drives GFP/Cre expression. Although this line has been widely used in the field, to our knowledge no separate primary publication specifically describing its original generation and full validation has been published. The ScxCre line was first used in the original study [10] and the Ca_V_1.2-TS^fl/fl^ [11] mouse line has been described previously.

3. Please include Jackson Laboratory strain numbers for all mouse lines used in the Methods.

**Thank you for this comment. We have revised the Methods section to include the Jackson Laboratory strain numbers for all mouse lines obtained from or currently available through The Jackson Laboratory.**

C57BL/6J (Strain No: 000664), RlacZ^fl/fl^ (Strain No: 003474), Ai9^fl/fl^ (Strain No: 007909) strains were obtained from the Jackson Laboratory. All mice were on a C57BL/6J genetic background.

4. Please verify references 12 and 15, as they appear potentially duplicated.

**Thank you for pointing this out. We have reviewed the reference list and removed the duplicated reference.**

**Reviewer #3:** The authors present a paper about dangers of incorrect breeding management regarding the widely used Scx-Cre mouse model, focusing on the dangers of germline recombination, when using male germline for propagation. In contrast, no spontaneous recombination was found when using female germline. This finding is an important information for adapting breeding strategies and colony management.
Although the paper is simple and technical, its findings are generally important and varrant publication.

If I were to suggest an improvement, the authors could further explore this finding in context of other, unrelated, transgene mouse strains and compare the situation, to better inform the reader of the broader context.

**Thank you for this positive assessment and helpful suggestion. We agree that placing our findings in the broader context of other Cre-driver mouse lines is important for readers. Unintended germline activity has been reported in multiple Cre-driver lines developed for tissue-specific recombination (PMID: 16419044, PMID:** [**18612081**](https://pubmed.ncbi.nlm.nih.gov/18612081/)**,** **PMID: 24349371, PMID: 32027825), indicating that this phenomenon is not unique to ScxCre. We did not perform a direct side-by-side comparison with another constitutive Cre line in the present study, but instead focused on comparing male and female ScxCre breeders. In addition, most Cre lines routinely available in our laboratory are tamoxifen-inducible CreER or CreERT2 lines, which differ substantially from constitutive ScxCre in their regulation and requirement for induction; therefore, they would not provide a directly comparable control for spontaneous germline recombination under the breeding conditions examined here. A systematic comparison across unrelated constitutive Cre lines would be valuable, but we believe this would be beyond the scope of the present study.**

Editor

Journal Requirements:

When submitting your revision, we need you to address these additional requirements.

1. Please ensure that your manuscript meets PLOS ONE's style requirements, including those for file naming. The PLOS ONE style templates can be found at [https://journals.plos.org/plosone/s/file?id=wjVg/PLOSOne_formatting_sample_main_body.pdf](https://urldefense.com/v3/__https:/journals.plos.org/plosone/s/file?id=wjVg*PLOSOne_formatting_sample_main_body.pdf__;Lw!!MkQhnT5yHH0MPvOc00c!WQGkxUmV5oQbNMu54RfV7R9zyWh_Hy9wUZFrNui7tiwAwTb5aT3c4Tl9msDLPvOrChBJadFRkLA1qzDbzokEnY8OSg$) and [https://journals.plos.org/plosone/s/file?id=ba62/PLOSOne_formatting_sample_title_authors_affiliations.pdf](https://urldefense.com/v3/__https:/track.editorialmanager.com/CL0/https:*2F*2Fjournals.plos.org*2Fplosone*2Fs*2Ffile*3Fid=ba62*2FPLOSOne_formatting_sample_title_authors_affiliations.pdf/1/010f019e62bed189-68789909-7408-4205-a0af-43b52601ea0e-000000/yw5yEZ_a4szqTC9rCbRd4spFws_77fIXazx8K9RhL5o=258__;JSUlJSUlJQ!!MkQhnT5yHH0MPvOc00c!WQGkxUmV5oQbNMu54RfV7R9zyWh_Hy9wUZFrNui7tiwAwTb5aT3c4Tl9msDLPvOrChBJadFRkLA1qzDbzomkIDnv5Q$)

**Thank you for the guidance. We have made the corresponding changes and revised the manuscript to comply with PLOS ONE’s style requirements, including formatting and file naming requirements.**

2. To comply with PLOS One submissions requirements, in your Methods section, please provide additional information regarding the experiments involving animals and ensure you have included details on (1) methods of sacrifice, (2) methods of anesthesia and/or analgesia, and (3) efforts to alleviate suffering.

**Thank you for this comment. We have revised the Methods section to include additional details regarding animal husbandry and euthanasia procedures. Specifically, we now state that adult mice were euthanized by CO₂ inhalation followed by confirmation of death by cervical dislocation, and that neonatal pups at P0–P4 were euthanized by hypothermia on ice, followed immediately by tissue collection. Because this study did not involve survival surgery or other invasive procedures requiring recovery, anesthesia and analgesia were not applicable.**

3. Please update your submission to use the PLOS LaTeX template. The template and more information on our requirements for LaTeX submissions can be found at [http://journals.plos.org/plosone/s/latex](https://urldefense.com/v3/__http:/track.editorialmanager.com/CL0/http:*2F*2Fjournals.plos.org*2Fplosone*2Fs*2Flatex/1/010f019e62bed189-68789909-7408-4205-a0af-43b52601ea0e-000000/_Bmo4aIwq5ouTZ8QkXZ5VC5SpAdDfUMr5MXEUvzeNT8=258__;JSUlJSU!!MkQhnT5yHH0MPvOc00c!WQGkxUmV5oQbNMu54RfV7R9zyWh_Hy9wUZFrNui7tiwAwTb5aT3c4Tl9msDLPvOrChBJadFRkLA1qzDbzokPbZcDCg$).

**We have updated our manuscript following the PlOS LaTeX template.**

4. Thank you for stating in your Funding Statement:

[This work was supported by NIH/NIAMS P30AR69655 and R01AR085590.].

Please provide an amended statement that declares *all* the funding or sources of support (whether external or internal to your organization) received during this study, as detailed online in our guide for authors at [http://journals.plos.org/plosone/s/submit-now](https://urldefense.com/v3/__http:/journals.plos.org/plosone/s/submit-now__;!!MkQhnT5yHH0MPvOc00c!WQGkxUmV5oQbNMu54RfV7R9zyWh_Hy9wUZFrNui7tiwAwTb5aT3c4Tl9msDLPvOrChBJadFRkLA1qzDbzomhmuLrdg$).  Please also include the statement “There was no additional external funding received for this study.” in your updated Funding Statement.

Please include your amended Funding Statement within your cover letter. We will change the online submission form on your behalf.

5. Please expand the acronyms “NIAMS” and “NIH” (as indicated in your financial disclosure) so that it states the name of your funders in full.

This information should be included in your cover letter; we will change the online submission form on your behalf.

6. Please remove any funding-related text from the manuscript. Please note that funding information should not appear in any section or other areas of your manuscript. We will publish the funding statement that is present in the Financial Disclosure section of the online submission form.

**For 4,5 and 6, we have amended the Funding Statement to declare all sources of support received for this study and have included the required statement: “There was no additional external funding received for this study” in the updated cover letter. We have removed the funding statement from the manuscript.**

7. PLOS ONE now requires that authors provide the original uncropped and unadjusted images underlying all blot or gel results reported in a submission’s figures or Supporting Information files. This policy and the journal’s other requirements for blot/gel reporting and figure preparation are described in detail at [https://journals.plos.org/plosone/s/figures#loc-blot-and-gel-reporting-requirements](https://urldefense.com/v3/__https:/journals.plos.org/plosone/s/figures*loc-blot-and-gel-reporting-requirements__;Iw!!MkQhnT5yHH0MPvOc00c!WQGkxUmV5oQbNMu54RfV7R9zyWh_Hy9wUZFrNui7tiwAwTb5aT3c4Tl9msDLPvOrChBJadFRkLA1qzDbzonmVptYKg$) and [https://journals.plos.org/plosone/s/figures#loc-preparing-figures-from-image-files](https://urldefense.com/v3/__https:/journals.plos.org/plosone/s/figures*loc-preparing-figures-from-image-files__;Iw!!MkQhnT5yHH0MPvOc00c!WQGkxUmV5oQbNMu54RfV7R9zyWh_Hy9wUZFrNui7tiwAwTb5aT3c4Tl9msDLPvOrChBJadFRkLA1qzDbzolIVm601g$). When you submit your revised manuscript, please ensure that your figures adhere fully to these guidelines and provide the original underlying images for all blot or gel data reported in your submission. See the following link for instructions on providing the original image data: [https://journals.plos.org/plosone/s/figures#loc-original-images-for-blots-and-gels](https://urldefense.com/v3/__https:/journals.plos.org/plosone/s/figures*loc-original-images-for-blots-and-gels__;Iw!!MkQhnT5yHH0MPvOc00c!WQGkxUmV5oQbNMu54RfV7R9zyWh_Hy9wUZFrNui7tiwAwTb5aT3c4Tl9msDLPvOrChBJadFRkLA1qzDbzok_shR5AA$).

In your cover letter, please note whether your blot/gel image data are in Supporting Information or posted at a public data repository, provide the repository URL if relevant, and provide specific details as to which raw blot/gel images, if any, are not available. Email us at plosone@plos.org if you have any questions.

If the reviewer comments include a recommendation to cite specific previously published works, please review and evaluate these publications to determine whether they are relevant and should be cited. There is no requirement to cite these works unless the editor has indicated otherwise.

**We have provided the original uncropped and unadjusted gel images in the Supporting Information files and have noted this in the revised cover letter.**

Additional Editor Comments:

Additional comments：
1， To help the readers to understand how the complicate mouse genotyping is performed, please draw detail figures showing the position of forward and reverse primers relating to specific genetic locus or targeting constructs.

**Thank you for this suggestion. We have added a detailed figure panel (Figure 1D) showing the positions and orientations of the forward and reverse primers relative to the corresponding Rosa26/Ai9 genetic loci and targeting constructs. The figure now includes the primer locations for the Rosa26 wild-type allele, the unrecombined Ai9 floxed allele, and the Cre-recombined Ai9Δ allele after deletion of the floxed STOP cassette.**

2, Please verify the accuracy of PCR primer sequences. A blast search showed a primer labeled as "RosaWT-R: 5’-CGTACTGACGGTGGGAGAAT-3" actually maps to "Cre recominase" coding sequence. Another blast search showed the primer labeled as "Ai9(floxed)-F: 5’-CTGTTCCTGTACGGCATGG-3’" matched to EGFP coding sequence. Please explain how the PCR is performed or correct it if there is a mistake.

**Thanks for pointing this out. We have carefully rechecked and corrected the primer sequence information for genotyping in the revised manuscript. For genotyping the Rosa 26 wildtype and Ai9 allele, we used the primer sequences recommended by the Jackson Laboratory for Ai9 mice (Jax stock no. 007909). The primers for the Cre-recombined Ai9Δ allele were developed based on the original publication (doi:10.1038/nn.2467).**
